# Hypothetical biomolecular probe based on a genetic switch with tunable symmetry and stability

**DOI:** 10.1186/s12918-016-0279-y

**Published:** 2016-06-06

**Authors:** Nikolay Martyushenko, Sigurd Hagen Johansen, Cheol-Min Ghim, Eivind Almaas

**Affiliations:** Department of Biotechnology, NTNU - Norwegian University of Science and Technology, Trondheim, N-7491 Norway; School of Life Sciences and Department of Physics, Ulsan National Institute of Science and Technology (UNIST), Ulsan, 44919 Korea; Mathematical Bioscience Institute, The Ohio State University, Columbus, 43210 USA

**Keywords:** Genetic toggle switch, Stochastic gene expression, LacI-TetR system, Nano-recorder

## Abstract

**Background:**

Genetic switches are ubiquitous in nature, frequently associated with the control of cellular functions and developmental programs. In the realm of synthetic biology, it is of great interest to engineer genetic circuits that can change their mode of operation from monostable to bistable, or even to multistable, based on the experimental fine-tuning of readily accessible parameters. In order to successfully design robust, bistable synthetic circuits to be used as biomolecular probes, or understand modes of operation of such naturally occurring circuits, we must identify parameters that are key in determining their characteristics.

**Results:**

Here, we analyze the bistability properties of a general, asymmetric genetic toggle switch based on a chemical-reaction kinetic description. By making appropriate approximations, we are able to reduce the system to two coupled differential equations. Their deterministic stability analysis and stochastic numerical simulations are in excellent agreement. Drawing upon this general framework, we develop a model of an experimentally realized asymmetric bistable genetic switch based on the LacI and TetR repressors. By varying the concentrations of two synthetic inducers, doxycycline and isopropyl *β*-D-1-thiogalactopyranoside, we predict that it will be possible to repeatedly fine-tune the mode of operation of this genetic switch from monostable to bistable, as well as the switching rates over many orders of magnitude, in an experimental setting. Furthermore, we find that the shape and size of the bistability region is closely connected with plasmid copy number.

**Conclusions:**

Based on our numerical calculations of the LacI-TetR asymmetric bistable switch phase diagram, we propose a generic work-flow for developing and applying biomolecular probes: Their initial state of operation should be specified by controlling inducer concentrations, and dilution due to cellular division would turn the probes into memory devices in which information could be preserved over multiple generations. Additionally, insights from our analysis of the LacI-TetR system suggest that this particular system is readily available to be employed in this kind of probe.

**Electronic supplementary material:**

The online version of this article (doi:10.1186/s12918-016-0279-y) contains supplementary material, which is available to authorized users.

## Background

Genetic switches and circuits are abundant in nature and control the regulation and genetic development programs that are at the basis of cellular function. Spanning simple feedback loops in *B. subtilis* competence switching [1] to complex differentiation patterns in humans [2], ultra-stable genetic switches present living organisms with a reliable mode of operation within a noisy state until a signal is received that triggers the transition to a different state of operation. The physical properties, dynamics and stability of genetic switches are important, not only because of their common occurrence in biological systems, but also for their potential use in synthetic biology in the engineering and design of more complicated cellular functions [3, 4].

An important challenge in the successful design of a genetic circuit is the fine-tuning of various circuit parameters [4]: In even simple genetic circuit topologies, it is most often necessary to fine-tune the strength and responsiveness of different circuit components in order to achieve intended functional behavior. For instance, the consequences of noise can significantly affect the function of a genetic circuit [5]. Thus, it is of great interest to develop circuits and components that are readily amenable to experimental control.

Among the early demonstrations of the engineering of synthetic circuits, Gardner et al. used molecular genetic tools to construct a bistable toggle switch consisting of two genes coding for mutually repressing proteins [6]. They also implemented control of the toggle-switch state of operation by using an inducer that could affect transcription in the circuit, and thus, affect its stability (monostable or bistable). Since then, a considerable amount of effort has been put into improving switch robustness, tunability and scalability. For instance Deans et al. combined tunability with robustness in a protein-RNA genetic switch [7], while Green et al. combined robustness with unprecedented scalability using an RNA toehold system [8]. While both protein and RNA switches are commonly used in scientific practice, the focus on robustness, being the lack of dynamics, has led to the fact that not many studies have investigated the dynamics of specific or generic genetic switches. Recently, however, Shopera et al. investigated the properties of several single and dual-positive feedback synthetic genetic switches, coupling experimental evidence to deterministic mathematical modelling, and amongst other things, generating experimental and theoretical stability diagrams [9]. Unfortunately, this study was limited by the absence of reliable kinetic information concerning the circuit system in question and by the lack of stochastic analysis, both experimental and theoretical.

Here, we discuss in detail a mathematical and computational model for a generic, asymmetric genetic toggle switch, following up on our previous reports on symmetric genetic switches [10, 11]. In these toggle switches, the active repressor species is a protein dimer. This class of switch was demonstrated to have a large dynamic range of switching frequency over a relatively small gene expression efficiency window [11].

At first we treat the general case, where the two mutually inhibiting genetic circuits are equivalent, except for a chosen set of kinetic constants making it asymmetric. For this circuit, we show that it is possible to reduce the system to two coupled non-linear differential equations that depend on only four tunable parameters (two for each gene). This is analogous to the analysis that was conducted in [11] with focus on a symmetric switch.

Next we use the framework of the general asymmetric toggle switch to develop a detailed mathematical model for an experimentally realizable switch. To make the model construct as accurate as possible, we elected to use transcription factors for which we can utilize the greatest amount of quantitative data. We decided to utilize the LacI and TetR repressors, which we will denote the LITR-switch. These repressors are especially interesting because they are amenable to fine-tuning by the addition of IPTG and doxycyline (inhibitors of lacI and TetR, and thus inducers of LacI and TetR-repressed expression respectively), allowing the degree of asymmetry caused by their differing expression strengths to be easily modified experimentally. Using values for kinetic constants derived from literature, we explored the circuit’s states of operation through both stochastic and deterministic simulations. Interestingly, some of our findings can be related to properties of the circuit previously studied in vivo by Gardner et al. [6], since one of their constructed toggle switches is quite similar in design.

## Results

**Fig. 1:**
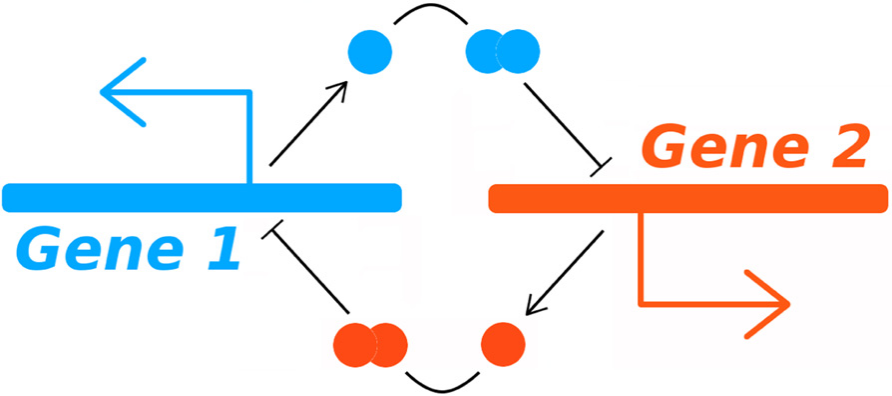
Schematic of asymmetric toggle switch. Promoter 1, D$^{1}_{ab}$, is the promoter for Gene 1, which encodes a repressor with specific binding for Promoter 2. Promoter 2, D$^{2}_{ab}$, is the promoter for Gene 2, which encodes a repressor with specific binding for Promoter 1. It may be possible to control the operation of the switch by the use of appropriate inducers

The generic bistable genetic switch is schematically depicted in Fig. 1: Two genes, each encoding a repressor, affect each other through homodimers. For gene 1, the promoter has two operator domains for repressor binding, and they are specific for the repressor encoded by gene 2. Additionally, we will assume that the homodimers will bind cooperatively at the two binding sites. The situation is similar for gene 2. For the mathematical model of a bistable switch with LacI-TetR repressors (the LITR switch) we additionally have access to two inducers.

A chemical reaction kinetic representation of the gene circuit processes is listed in Table 1. Here, the promoter controlling the expression of gene *l* is denoted by D$^{l}_{ij}$, and the index *i* corresponds to the number of repressors bound at the operator site (allowed states being 0,1 or 2). The index *j* accounts for the RNA polymerase (R) binding states (0 - unbound, 1 - bound) of the promoter. E^*l*^ describes the state when R is bound to the DNA but has cleared the promoter, leading to the transcription of mRNA (M^*l*^). This is followed by translation to produce monomer proteins (P^*l*^), which are allowed to form homodimers (P$_{2}^{l}$). For the reversible reactions, we use *k*_*lm*_ for the forward and *q*_*lm*_ for the reverse rates, where *m* indicates the reaction number (see Table 1).

**Table 1:** Processes included in the HOM2 genetic circuit description

Type of reaction	Gene 1	Gene 2
Repressor binding	$\mathrm {D}^{1}_{00} + \mathrm {P}^{2}_{2} \underset {q_{12}}{\overset {k_{12}}{\rightleftharpoons }} \mathrm {D}^{1}_{10}$	$\mathrm {D}^{2}_{00} + \mathrm {P}^{1}_{2} \underset {q_{22}}{\overset {k_{22}}{\rightleftharpoons }} \mathrm {D}^{2}_{10}$
	$\mathrm {D}^{1}_{10} + \mathrm {P}^{2}_{2} \underset {q_{14}}{\overset {k_{14}}{\rightleftharpoons }} \mathrm {D}^{1}_{20}$	$\mathrm {D}^{2}_{10} + {P^{1}_{2}} \underset {q_{24}}{\overset {k_{24}}{\rightleftharpoons }} \mathrm {D}^{2}_{20}$
RNAp binding	$\mathrm {D}^{1}_{00} + \mathrm {R} \underset {q_{13}}{\overset {k_{13}}{\rightleftharpoons }} \mathrm {D}^{1}_{01}$	$\mathrm {D}^{2}_{00} + \mathrm {R} \underset {q_{23}}{\overset {k_{23}}{\rightleftharpoons }} \mathrm {D}^{2}_{01}$
	$\mathrm {D}^{1}_{10} + \mathrm {R} \underset {q_{15}}{\overset {k_{15}}{\rightleftharpoons }} \mathrm {D}^{1}_{11}$	$\mathrm {D}^{2}_{10} + \mathrm {R} \underset {q_{25}}{\overset {k_{25}}{\rightleftharpoons }} \mathrm {D}^{2}_{11}$
	$\mathrm {D}^{1}_{20} + \mathrm {R} \underset {q_{17}}{\overset {k_{17}}{\rightleftharpoons }} \mathrm {D}^{1}_{21}$	$\mathrm {D}^{2}_{20} + \mathrm {R} \underset {q_{27}}{\overset {k_{27}}{\rightleftharpoons }} \mathrm {D}^{2}_{21}$
Transcription initiation	$\mathrm {D}^{1}_{01} \overset {\alpha _{1m}}{\rightarrow } \mathrm {E}^{1} + \mathrm {D}^{1}_{00}$	$\mathrm {D}^{2}_{01} \overset {\alpha _{2m}}{\rightarrow } \mathrm {E}^{2} + \mathrm {D}^{2}_{00}$
	$\mathrm {D}^{1}_{11} \overset {\alpha _{1m}}{\rightarrow } \mathrm {E}^{1} + \mathrm {D}^{1}_{10}$	$\mathrm {D}^{2}_{11} \overset {\alpha _{2m}}{\rightarrow } \mathrm {E}^{2} + \mathrm {D}^{2}_{10}$
	$\mathrm {D}^{1}_{21} \overset {\alpha _{1m}}{\rightarrow } \mathrm {E}^{1} + \mathrm {D}^{1}_{20}$	$\mathrm {D}^{2}_{21} \overset {\alpha _{2m}}{\rightarrow } \mathrm {E}^{2} + \mathrm {D}^{2}_{20}$
Elongation	$\mathrm {E}^{1} \overset {\alpha _{1m}^{\prime }}{\rightarrow } \mathrm {M}^{1} + \mathrm {R} $	$\mathrm {E}^{2} \overset {\alpha _{2m}^{\prime }}{\rightarrow } \mathrm {M}^{2} + \mathrm {R} $
Translation	$\mathrm {M}^{1} \overset {\alpha _{1p}}{\rightarrow } \mathrm {P}^{1} + \mathrm {M}^{1} $	$\mathrm {M}^{2} \overset {\alpha _{2p}}{\rightarrow } \mathrm {P}^{2} + \mathrm {M}^{2} $
Dimerization	$\mathrm {P}^{1} + \mathrm {P}^{1} \underset {q_{11}}{\overset {k_{11}}{\rightleftharpoons }} \mathrm {P}^{1}_{2}$	$\mathrm {P}^{2} + \mathrm {P}^{2} \underset {q_{21}}{\overset {k_{21}}{\rightleftharpoons }} \mathrm {P}^{2}_{2}$
Degradation	$\mathrm {M}^{1} \overset {\gamma _{1m}}{\rightarrow } \oslash $	$\mathrm {M}^{2} \overset {\gamma _{2m}}{\rightarrow } \oslash $
	$\mathrm {P}^{1} \overset {\gamma _{1p}}{\rightarrow } \oslash $	$\mathrm {P}^{2} \overset {\gamma _{2p}}{\rightarrow } \oslash $
	$\mathrm {P}^{1}_{2} \overset {\gamma _{1p}/\sigma _{1}}{\rightarrow } \oslash $	$\mathrm {P}^{2}_{2} \overset {\gamma _{2p}/\sigma _{2}}{\rightarrow } \oslash $

In the first column, the reaction class is described in words. The reaction rates are written above or below the arrows describing the forward and reverse rate respectively

Under the “adiabatic” approximation that all the binding-unbinding reactions are in equilibrium, the resultant dynamics for the active transcription factor level, *T*_1_ and *T*_2_, is given by the following set of coupled nonlinear differential equations:

1 $$ \begin{aligned} \left. \begin{array}{ll} \frac{\mathrm{d}T_{1}}{\mathrm{d}t} = 2\sqrt{\theta_{1} T_{1}} \lambda_{1}\left(1+\frac{\nu_{1}}{1 + \mu_{1} T_{2}~(1 + r_{1} T_{2})}\right) -2\left(T_{1} + \frac{2}{\kappa_{1} \sqrt{\theta_{1}}} T_{1}^{3/2}\right)\\ \frac{\mathrm{d}T_{2}}{\mathrm{d}t}= 2\sqrt{\theta_{2} T_{2}} \lambda_{2}\left(1+\frac{\nu_{2}}{1 + \mu_{2} T_{1}~(1 + r_{2} T_{1})}\right) -2\left(T_{2} + \frac{2}{\kappa_{2} \sqrt{\theta_{2}}} T_{2}^{3/2}\right) \end{array} \right\} \end{aligned}  $$

where the parameters are all defined in the Methods section. The form of Eq. (1) lends itself to the following interpretation: Since the first term is always positive, we may consider it an effective synthesis rate in a birth-death process. The second term is always negative and would correspond to the decay rate in such a process. The synthesis term of a dimer is proportional to the root of its own concentration, since the dimer must be produced from the monomer and equilibrium is assumed to occur instantaneously. The first part of this term corresponds to protein synthesis at full repression, while the second represents repression by the competing agent. Furthermore, the first part of the decay term represents degradation of dimers, while the second represents that of the monomers. This result is the generalization of the symmetric HOM2 circuit in Ghim and Almaas (2009) [11] to the situation where the two interacting genes have different characteristics.

In the calculations culminating in Eq. (1), we have applied approximations (i)-(iv) described in the Methods section and the simplification that the dissociation parameters *K*_*l*7_=*K*_*l*5_. We do not believe that these significantly reduce the generality of Eq. (1). The main assumption here is the adiabatic assumptions (ii) and (iv), that the repressor binding and dimerization as well as transcriptional elongation reactions are in steady state. While making the problem analytically tractable, this assumption has been shown to have only minor effects on the results of genetic switch simulations [11]. The mentioned simplification of the two dissociation parameters equates the RNApol binding constants in a promoter singly and doubly bound by repressors. Though this is not the case in a general system, the simplification still preserves the effect of double repressor binding. Even thought the binding of the second repressor does not affect transcription initiation efficiency directly, it reduces the probability of a repressor-free promoter, thus thereby still reducing the average transcription rate. Additionally, when exploring the properties of the system, we have made the simplifying assumptions *γ*_*lp*_=*γ*_*p*_ and *K*_*l*2_=*K*_2_. The first assumption equates the protein degradation rates of the two switch-components. This is true for E.*coli* in exponential growth phase, as protein degradation in that case is dominated by dilution due to cell division [12]. The final assumption is that the affinities of the two repressor types for their respective promoters are the same. This assumption was implemented in order to restrict the exploration of the system to only two parameters, as seen in the next section.

### Reduced system: deterministic and stochastic analysis

We solve Eq. (1) numerically for the steady state at different parameter values, and we identified the number of *stable* steady states. The parameters that we choose to vary are *s* and *β*, which take part in parameters *ν*,*μ* and *λ* of Eq. (1). It can be seen in the Methods section that *s* corresponds to promoter leakage, while *β* represents gene expression efficiency. Unless otherwise noted, the parameter values used in this null cline analysis are the same as in [11] (based on the CI repressor from bacteriophage *λ*) and as listed in Table 2. These parameter values will also serve as a bistable reference point in the *s*−*β* phase plane.

**Table 2:** Parameter values used to perform a deterministic analysis of the general, reduced system based on selections made in [11]

*K*_2_	*K*_*l*1_	*r*_*l*_	*s*_*l*_	*β*_*l*_	*u*_*l*_	*σ*_*l*_
20 nM	10 nM	25	0.01	17.5 nM^−1^	3.0	10

When the parameter index is given as *l*, the same value is chosen for both gene 1 and 2

**Fig. 2:**
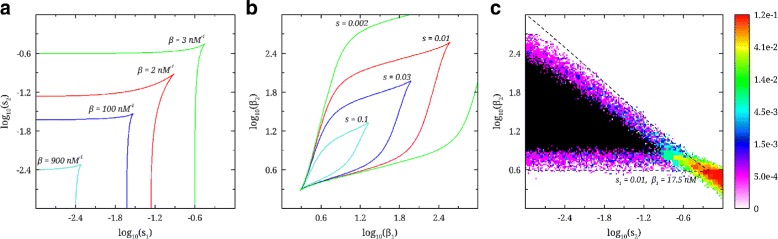
Deterministic stability diagrams for generic asymmetric switch. **a** *s*_1_ vs. *s*_2_ for varying values of *β*_1_=*β*_2_=*β*={2,3,100,900}(nM^−1^). Bistability region is inside the curves. **b** *β*_1_ vs. *β*_2_ for varying values of *s*_1_=*s*_2_=*s*={0.002,0.01,0.03,0.1}. Bistability region is enclosed by the curves. **c** *s*_2_ vs. *β*_2_. *s*_1_ and *β*_1_ are held at 0.01 and 17.5 respectively. The *dotted black line* encloses the deterministically determined bistable region. A stochastically determined joint bistable region (bistable with switching) is represented as as heat plot in colors from *purple* to *red*. The *heat bar* represents the switching frequency in Hz. The *black region* in the middle represents bistability where no switching occurred for the duration of the simulation of (2×10^3^ seconds)

We start exploring the effect of circuit parameter asymmetry on the stability of the toggle switch by changing pairs of similar parameters describing the two genes. Figure 2(a) shows the consequences of varying the leakage from each of the promoters, described by the parameters *s*_1_ and *s*_2_, for a sequence of gene expression efficiencies *β*={2,3,17.5,100,900}(nM^−1^). The *s*_*l*_ parameters can take values independent of each other, and to a quite high extent, their values may be modified by genetic manipulation. Since all the parameters, except from *s*_1_ and *s*_2_, are chosen to be identical in the two genes, the symmetry around the diagonal in the plot is to be expected, and thus serves as an internal consistency check on our calculations. We note a non-linear change in the shape of the bistability region in response to changing *β*: As *β* is decreasing towards *β*∼3 nM^−1^, the bistable region is expanding. However, further decreases in *β* lead to a sharp contraction of the bistable region.

The individual gene expression efficiency from each of the promoters, described by the parameters *β*_1_ and *β*_2_, are able to vary independently of each other. The *β*-variables can potentially be modified by genetic manipulation, especially by modifying the corresponding 5’UTR regions [13–15], making the *β*_1_ vs *β*_2_ stability diagram of great interest. Figure 2(b) displays the region of bistability (inside the curves) for the sequence of values *s*={0.002,0.01,0.03,0.1}. As promoter leakage *s* increases, the bistable region shows a systematic decrease.

Figure 2(c) shows the stability diagram (dotted black line) when varying *β*_2_ and *s*_2_ while keeping *β*_1_ and *s*_1_ as in Table 2. We note that, while the general shape is similar to that found for the simultaneous and identical change of *β*_*l*_ and *s*_*l*_ [11], the extremal part of the bistability region (large *s*_2_) is much sharper in the current case.

We will later simulate the full reaction set of the LITR-system, as described by Eqs. (A.1)-(A.21) and, for the sake of brevity, do not present such simulations of the current circuit. Previously, we employed an approximate representation of the stochastic dynamics for the symmetric version of the circuit [11]: Assuming a simple birth-death process where the propensities for synthesis and decay are described by Eq. (1), this stochastic system is governed by the average rates we calculated under the adiabatic assumptions (i)-(iv), which we detail in the Methods section.

We also conduct a stochastic exploration of the stability diagram (demarcated by dotted lines) reported in Fig. 2(c) by keeping the pair of the leakage/efficiency parameters at their reference values, *s*_1_=0.01*β*_1_=17.5 nM^−1^, while varying *s*_2_ and *β*_2_ as indicated by the axes in Fig. 2(c). The bistable gene-switch system has two possible starting points: either gene 1 is in an active state (with high protein copy number) or in an inactive state (with low protein copy number), and we use both conditions as starting points for two separate 10^3^ time-step and 10^7^ time-step simulations.

The results reported in Fig. 2(c) show that the bistable region determined by stochastic simulations lies within, and almost entirely fills, the one calculated using deterministic analysis. The switching rate is not of great practical relevance here, because the time is scaled relative to that of the protein decay rate. The high switching rate region that extends out of the deterministic bistable region corresponds to a situation where the two genes behave independently of one another (see next section for more detail).

### LITR system: stochastic analysis

Following our analysis of the generalized reduced genetic switch, we decided to develop a realistic model for a switch that could be experimentally implemented. We simulated the circuit and looked at its dynamic properties.

For this we needed to identify two transcription factors for which enough kinetic data is available and which state of operation could be tunable with small molecule inducers. The tunability is necessary to make the circuit accessible to experimental manipulation for mapping out its stability states. The choice of small molecule inducers for the circuit manipulation (as opposed to e.g. promoter sequence alterations or genetic mutations of the repressor species) was to make the in vivo experimental system tunable and possible to re-set.

Thus, we selected to analyze a genetic switch composed of the LacI and TetR factors and their non-metabolizable inducers, isopropyl *β*-D-1-thiogalactopyranoside (IPTG) and doxycycline (DOX) respectively. In our model of the LacI-TetR bistable genetic switch, the LITR system, we explicitly include modules for the DNA binding of these transcription factors, based on literature information (see Methods for details). A literature search resulted in numeric values for 30 different kinetic parameters to support our model development (see Methods for details). As a result, the number of species in our model description grew from 20 for the generic asymmetric circuit to 30 for LITR, and the number of reactions increased considerably. The increase in complexity was largely due to the addition of small-molecule bound species, e.g. LacI-IPTG complexes, and their corresponding reactions. Note that, adding IPTG or DOX would result in effective changes in the definition of *β* and *s* parameters of the reduced system. Moreover, LacI was found to be active mostly in its tetrameric form, which can bind two different sites at once on the DNA, unlike the TetR dimer that only can bind a single site. This makes the switch asymmetric both by the values of kinetic constants and by the number and type of reactions that are involved. A list of all reactions in the LITR switch is shown in Table 3, while a summary of the parameters we obtained can be found in Table 4.

**Table 3:** Processes included in the LacI-TetR (LITR) genetic circuit

Type of reaction	*lacI* (with P_tet_)	*tetR* (with P_lac_)
Repressor binding	$\text {TetR}_{2} + \mathrm {D}_{l} \underset {q_{t3}}{\overset {k_{t3}}{\rightleftharpoons }} \mathrm {d}_{1}\text {TetR}_{2}$	$\text {LacI}_{4} + \mathrm {D}_{t} \underset {q_{l3}}{\overset {k_{l3}}{\rightleftharpoons }} \mathrm {d}_{1}\text {LacI}_{4}$
	$\mathrm {d}_{1}\text {TetR}_{2} + \mathrm {D}_{l} \underset {q_{t3}}{\overset {k_{t3}}{\rightleftharpoons }} \mathrm {d}_{2}\text {TetR}_{2}$	$\text {LacI}_{4i} + \mathrm {D}_{t} \underset {q_{l3}}{\overset {0.5k_{l3}}{\rightleftharpoons }} \mathrm {d}_{1}\text {LacI}_{4i}$
	$\mathrm {d}_{1}\text {TetR}_{2} + \mathrm {I}_{t} {\overset {k_{t2}}{\rightarrow }} \text {TetR}_{2i} + \mathrm {D}_{l}$	$\mathrm {d}_{1}\text {LacI}_{4} \underset {q_{l4}}{\overset {200k_{l3}}{\rightleftharpoons }} \mathrm {d}_{2}\text {LacI}_{4}$
	$\mathrm {d}_{2}\text {TetR}_{2} + \mathrm {I}_{t} {\overset {k_{t2}}{\rightarrow }} \text {TetR}_{2i} + \mathrm {d}_{1}\text {TetR}_{2}$	$\mathrm {d}_{1}\text {LacI}_{4i} + \mathrm {I}_{l} {\overset {k_{l5}}{\rightarrow }} \text {LacI}_{4ii} + \mathrm {D}_{t}$
		$\mathrm {d}_{1}\text {LacI}_{4} + \mathrm {I}_{l}{\overset {0.5k_{l5}}{\rightarrow }} \text {LacI}_{4i} + \mathrm {D}_{t}$
		$\mathrm {d}_{1}\text {LacI}_{4} + \mathrm {I}_{l}\underset {q_{l5}}{\overset {0.5k_{l5}}{\rightleftharpoons }} \mathrm {d}_{1}\text {LacI}_{4i}$
		$\mathrm {d}_{2}\text {LacI}_{4} + \mathrm {I}_{l}\underset {q_{l5}}{\overset {k_{l5}}{\rightleftharpoons }} \mathrm {d}_{1}\text {LacI}_{4i}$
RNAp binding	$\mathrm {D}_{l} + \mathrm {R} \underset {q_{b}}{\overset {k_{b1}}{\rightleftharpoons }} \mathrm {D}^{\mathrm {R}}_{l}$	$\mathrm {D}_{t} + \mathrm {R} \underset {q_{b}}{\overset {k_{b1}}{\rightleftharpoons }} \mathrm {D}^{\mathrm {R}}_{t}$
	$\mathrm {d}_{1}\text {LacI}_{4} + \mathrm {R} \underset {q_{b}}{\overset {k_{lb2}}{\rightleftharpoons }} \mathrm {d}_{1}\text {LacI}^{\mathrm {R}}_{4}$	$\mathrm {d}_{1}\text {TetR}_{2} + \mathrm {R} \underset {q_{b}}{\overset {k_{tb2}}{\rightleftharpoons }} \mathrm {d}_{2}\text {TetR}^{\mathrm {R}}_{2}$
	$\mathrm {d}_{2}\text {LacI}_{4} + \mathrm {R} \underset {q_{b}}{\overset {k_{lb3}}{\rightleftharpoons }} \mathrm {d}_{2}\text {LacI}^{\mathrm {R}}_{4}$	$\mathrm {d}_{2}\text {TetR}_{2} + \mathrm {R} \underset {q_{b}}{\overset {k_{tb3}}{\rightleftharpoons }} \mathrm {d}_{2}\text {TetR}^{\mathrm {R}}_{2}$
	$\mathrm {d}_{1}\text {LacI}_{4i} + \mathrm {R} \underset {q_{b}}{\overset {k_{lb2}}{\rightleftharpoons }} \mathrm {d}_{1}\text {LacI}^{\mathrm {R}}_{4i}$	
Transcription initiation	$\mathrm {D}^{\mathrm {R}}_{l} \overset {\alpha _{i}}{\rightarrow } \mathrm {E}^{1} + \mathrm {D}_{l}$	$\mathrm {D}^{\mathrm {R}}_{t} \overset {\alpha _{i}}{\rightarrow } \mathrm {E}^{2} + \mathrm {E}_{t}$
	$\mathrm {d}_{1}\text {LacI}^{\mathrm {R}}_{4} \overset {\alpha _{i}}{\rightarrow } \mathrm {E}^{1} + \mathrm {d}_{1}\text {LacI}_{4}$	$\mathrm {d}_{2}\text {TetR}^{\mathrm {R}}_{2} \overset {\alpha _{i}}{\rightarrow } \mathrm {E}^{2} + \mathrm {d}_{2}\text {TetR}_{2}$
	$\mathrm {d}_{2}\text {LacI}^{\mathrm {R}}_{4} \overset {\alpha _{i}}{\rightarrow } \mathrm {E}^{1} + \mathrm {d}_{2}\text {LacI}_{4}$	$\mathrm {d}_{2}\text {TetR}^{\mathrm {R}}_{2} \overset {\alpha _{i}}{\rightarrow } \mathrm {E}^{2} + \mathrm {d}_{2}\text {TetR}_{2}$
	$\mathrm {d}_{1}\text {LacI}^{\mathrm {R}}_{4i} \overset {\alpha _{i}}{\rightarrow } \mathrm {E}^{1} + \mathrm {d}_{1}\text {LacI}_{4i}$	
Elongation	$\mathrm {E}_{l} \overset {\alpha _{le}}{\rightarrow } \mathrm {M}_{l} + \mathrm {R} $	$\mathrm {E}_{t} \overset {\alpha _{te}}{\rightarrow } \mathrm {M}_{t} + \mathrm {R} $
Translation	$\mathrm {M}_{l} \overset {\alpha _{lt}}{\rightarrow } \text {LacI} + \mathrm {M}_{l} $	$\mathrm {M}_{t} \overset {\alpha _{tt}}{\rightarrow } \text {TetR} + \mathrm {M}_{t} $
Dimerization	$\text {LacI} + \text {LacI} \underset {q_{1}}{\overset {k_{1}}{\rightleftharpoons }} \text {LacI}_{2}$	$\text {TetR} + \text {TetR} \underset {q_{1}}{\overset {k_{1}}{\rightleftharpoons }} \text {TetR}_{2}$
	$\text {LacI}_{2} + \text {LacI}_{2} \underset {q_{l2}}{\overset {k_{1}}{\rightleftharpoons }} \text {LacI}_{4}$	
Repressor Binding	$\text {LacI}_{4} + \mathrm {I}_{l} \underset {q_{l5}}{\overset {k_{l5}}{\rightleftharpoons }} \text {LacI}_{4i}$	$\text {TetR}_{2} + \mathrm {I}_{t} \underset {q_{t2}}{\overset {k_{t2}}{\rightleftharpoons }} \text {TetR}_{2i}$
	$\text {LacI}_{4i} + \mathrm {I}_{l} \underset {q_{l5}}{\overset {k_{l5}}{\rightleftharpoons }} \text {LacI}_{4ii}$	$\text {TetR}_{2i} + \mathrm {I}_{t} \underset {q_{t2}}{\overset {k_{t2}}{\rightleftharpoons }} \text {TetR}_{2ii}$
Degradation	$\mathrm {M}_{l} \overset {\gamma _{ml}}{\rightarrow } \oslash $	$\mathrm {M}_{t} \overset {\gamma _{mt}}{\rightarrow } \oslash $
	$\text {LacI} \overset {\gamma _{p}}{\rightarrow } \oslash $	$\text {TetR} \overset {\gamma _{p}}{\rightarrow } \oslash $
	$\text {LacI}_{2} \overset {\gamma _{p}}{\rightarrow } \oslash $	$\text {TetR}_{2} \overset {\gamma _{p}}{\rightarrow } \oslash $
	$\text {LacI}_{4} \overset {\gamma _{p}}{\rightarrow } \oslash $	$\text {TetR}_{2i} \overset {\gamma _{p}}{\rightarrow } \oslash $
	$\text {LacI}_{4i} \overset {\gamma _{p}}{\rightarrow } \oslash $	$\text {TetR}_{2ii} \overset {\gamma _{p}}{\rightarrow } \oslash $
	$\text {LacI}_{4ii} \overset {\gamma _{p}}{\rightarrow } \oslash $	

The rate constants are written above and below the arrows describing the forward and reverse rate respectively

**Table 4:** Summary of the parameter values used to perform stochastic and deterministic analysis of the realistic LacI-TetR system, based on the sources described in Methods

*α*_*le*_	0.065 s^−1^	*α*_*te*_	0.11 s^−1^	*α*_*i*_	0.66 s^−1^
*α*_*lt*_	0.01 s^−1^	*α*_*tt*_	0.017 s^−1^	*γ*_*ml*_	3.0 ×10^−3^s^−1^
*γ*_*mt*_	3.9 ×10^−3^s^−1^	*γ*_*p*_	3.9 ×10^−4^s^−1^	*k*_1_	0.015 *μ*m^3^
*q*_1_	8.8 ×10^−5^s^−1^	*q*_*l*2_	8.8 ×10^−8^s^−1^	*k*_*t*2_	2.4 ×10^−4^ *μ*m^3^
*q*_*t*2_	0.019 s^−1^	*k*_*l*3_	3.0 ×10^−3^ *μ*m^3^	*q*_*l*3_	5.4 ×10^−7^s^−1^
*k*_*t*3_	4.8 ×10^−3^ *μ*m^3^	*q*_*t*3_	2.1 ×10^−3^s^−1^	*q*_*l*4_	3.0 ×10^−3^s^−1^
*k*_*l*5_	1.4 ×10^−6^ *μ*m^3^	*q*_*l*5_	8.2 ×10^−3^s^−1^	*k*_*b*1_	4.3 ×10^−3^ *μ*m^3^
*q*_*b*_	0.45 s^−1^	*k*_*lb*2_	1.2 ×10^−5^ *μ*m^3^	*k*_*tb*2_	5.7 ×10^−6^ *μ*m^3^
*k*_*lb*3_	3.6 ×10^−7^ *μ*m^3^	*k*_*tb*3_	8.6 ×10^−6^ *μ*m^3^		

The indices*l*and *t* refer to LacI and TetR respectively. If this letter index is not given, the parameter is chosen to be the same for both species

We decided to investigate the stability of the circuit from a stochastic perspective. To this end, we simulated the full system for time-courses of 10^7^ s, each implemented with a state sampling every second. We ran two independent simulations with initial conditions being either 400 LacI tetramers or 400 TetR dimers, all other proteins and mRNA species numbers set to zero. We determined the stability by looking at the distributions of *Δ*=[LacI_4_]−[TetR_2_] over the time course of the simulation assuming three possible stability scenarios: (i) Monostability would occur if, for both initial conditions, the switch would be turned to the same side with overwhelming probability. (ii) Bistability would be determined if the initial conditions gave rise to two non-intersecting distributions of *Δ*. Finally, (iii) the *Δ* distribution could be bistable but with switching occurring between the two states (*joint bistable* case).

**Fig. 3:**
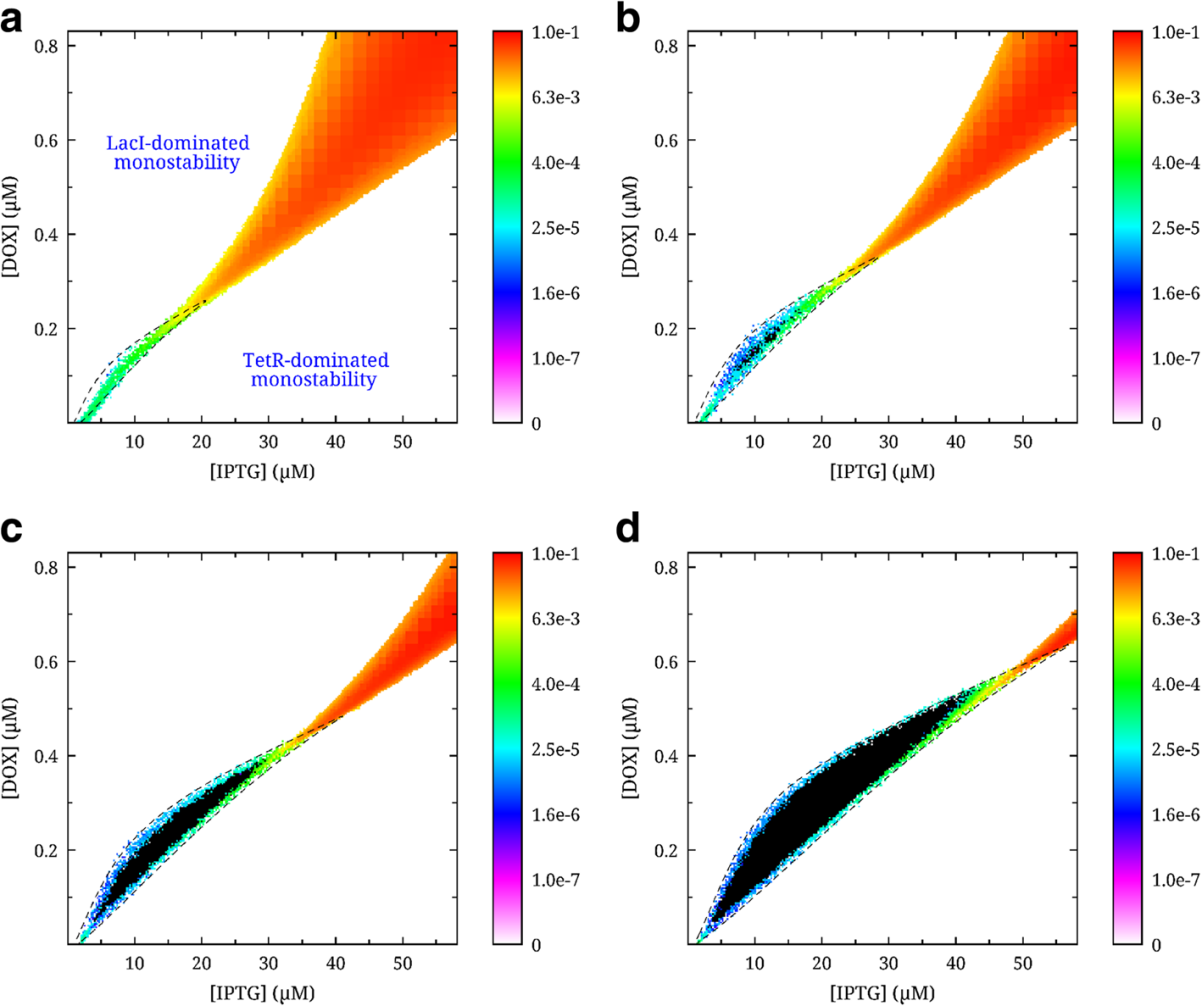
Stability in stochastic and deterministic simulations of the LacI-TetR (LITR) genetic switch. The *stability plots* represent the same genetic circuit with different plasmid copy numbers, ie. the total copy number of LacI and TetR genes: **a** 1 copy, **b** 2 copies, **c** 4 copies, and **d** 8 copies. Monostable regions are represented in *white*. The *dotted black line* encloses the deterministically determined bistable regions. Joint bistable regions (bistable with switching) are represented as heat plots in colors *from* purple to *red*. The *heat bars* represent the switching frequency in Hz. *Black regions* represent bistable regions where no switching occurred for the duration of the simulation time (10^7^ seconds)

We investigated the stability of our circuit as a function of [IPTG] and [DOX], as well as of plasmid copy number. The results can be seen in Fig. 3: Given just one copy of each gene, the stochastic simulations did not identify any disjoint bistability, ie. there was always switching between the two states: The slowest switching between the two possible states occurs at low inducer concentrations, with a frequency corresponding to a time scale of days. At a plasmid copy number of two (Fig. 3(b)) a small bistable region appears (black), and the size of the region of disjoint bistability increases with plasmid copy number. This bistable region appears to be within the range of [IPTG] and [DOX], where changes in their concentrations have experimentally been shown to bring about changes in transcription rates [16, 17].

In all cases, we observe a joint bistable region with high switching rate at large inducer concentrations. In this region, the number of transcription factors not bound to inducers is very close to zero. Consequently, the lack of regulation makes the two genes virtually decoupled and constitutively expressed. Also, it appears that LacI is a more potent transcriptional repressor than TetR, because at zero [IPTG] and [DOX] the system is monostable, with lacI being the “winning” species. This agrees well with experimental observations of Gardner et al. [6]. Here, LacI was expressed under the control of a weaker promoter (P_LtetO-1_) than the TetR (P_trc_) in the LITR circuit. Consequently, it was necessary to further reduce its strength in order to experimentally observe bistability. In our case, both species are expressed under the same P_R_ promoter, which should give rise to strong monostability in the absence of inducers. It is worth noting that in these simulations, we use the LacI binding operator O^sym^, which is much stronger than O^1^ used by Gardner et al. Nevertheless, when we conducted simulations of the system with appropriately reduced operator strength to explore this effect (not shown), the results showed only minor deviations. In this case we used the binding constant for the O^1^ operator taken from Garcia et al. [18]. We also observed that, if the copy number of *tetR* gene was set to four times that of *lacI*, the LITR system becomes bistable at zero inducer concentration (not shown). Thus the relative gene copy numbers could be used as a separate parameter to tune the symmetry of genetic switches.

**Fig. 4:**
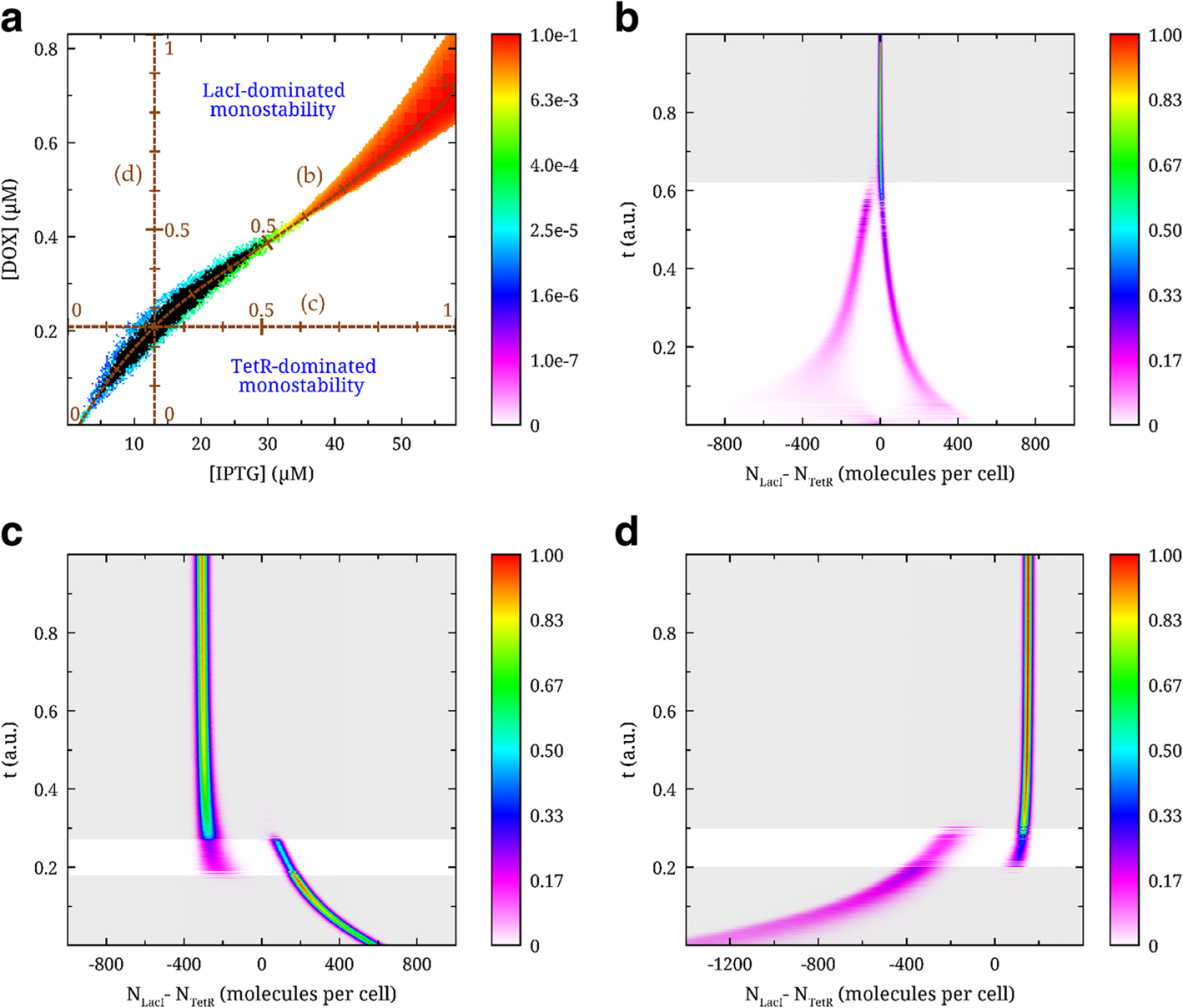
Stochastic simulations along three trajectories in inducer concentration space. **a** Schematic plot of three trajectories parametrized using the parameter 0 ≤*t*≤1, ie. *t* is the fractional displacement traversed by a trajectory. **b** Histogram of the trajectory along and inside of the bistable region. **c** Plot along the horizontal trajectory of increasing [IPTG]. **d** Plot along the vertical trajectory of increasing [DOX]. Each histogram bin corresponds to the normalized count that the difference between the numbers of the LacI and TetR active species had a particular value during the time course of the simulation (10^7^ s). The histograms are horizontal and the bin count represented with colour on a linear scale, normalized to the largest bin among all histograms on the plot. The y-axis represents *t*, the position along the trajectory. The areas tinted *grey* indicate regions of monostability, while the non-tinted indicate regions of true bistability

An interesting practical application of these observations would be to initialize the switch into a desired state, or to randomize its state, by altering the inducer concentrations (red regions in Fig. 3). In Fig. 4 we display histograms of *Δ* for three different titration schemes, depicted in panel (a): Panel (b), a titration scheme in which the [IPTG] and [DOX] are increased simultaneously, and panels (c) and (d), where the concentration of either inducer is increased independently. In Fig. 4(c,d) we find that the protein copy numbers realized in the bistable region depend on the monostable state from which this region is approached. Thus, the joint bistable region that surrounds disjoint bistability corresponds to the situation where one of the potential wells is shallow while the other is deep.

A dilution experiment starting from any point in the diagram in Fig. 4(a) would result in an [IPTG]-[DOX] phase-space movement along a straight line towards the origin. Thus, the switch could keep both its state and bistability upon dilution if the stability diagram would be more symmetric than what is shown here. A symmetrization of the bistability phase plot could be achieved for this system by adjusting plasmid copy numbers (see above). The histogram from running the system along a titration trajectory near the diagonal in Fig. 4(a) can be seen in Fig. 4(b). Similarly, if [IPTG] and [DOX] would be set to large values within the fast-switching bistable region of Fig. 4(a), a dilution would result in a (biased) randomization of the switch values that would depend on the starting concentrations of the inducers.

### LITR system: deterministic analysis

In order to investigate the agreement between stochastic modelling and deterministic analysis, we performed a deterministic stability analysis of the LITR system. The results of the analysis are given in Fig. 3. Here, the borders of the deterministically determined bistable regions are displayed as dashed black lines. It can be seen that the bistable regions of the stochastic simulation lie within the boundaries determined deterministically with the exception of the circuit with just one copy of each gene (Fig. 3(a)). Interestingly, the stochastic simulation in this case shows LacI to be a stronger transcriptional repressor than in the deterministic analysis: In panel Fig. 3(a), we see that the bistability region is shifted towards larger values of [IPTG], and it is especially evident for [DOX] ∼0. This effect is likely explained by a combination of the shallowness of the wells in the potential landscape at small inducer concentrations and our definition of bistability: The definition is based on the time that the system spends with [LacI_4_]>[TetR_2_] and vice versa, not on the shape of the potential landscape. This is because the potential landscape can be rugged, which increases the likelihood of detecting false wells. At larger gene copy numbers, stochastic and deterministic simulations agree very well. This is likely owing to the increased depth of the potential wells in that case. Also, the deterministic analysis does not identify the stochastically determined fast switching bistable region (red) as bistable. This is again related to our definition of bistability for the stochastic simulation. In this case, the potential well is very narrow and centered at zero (see Fig. 4b).

## Discussion

In this article we have analyzed and outlined the workings of an asymmetric genetic switch. At first, we looked at a generalized example of a bistable genetic switch. While it was relatively straightforward to simplify and theorize, it was challenging to relate this specific circuit implementation to experimental realizations, because its asymmetry-controlling parameters are difficult to access directly in an experimental setting. Therefore, we conducted an investigation of a realistic circuit, the LITR system, based on a mutually repressing *LacI*-*TetR* design. While this is challenging to simplify, the computational time needed for direct stochastic and deterministic simulations of this system is not prohibitive. Since we use small molecules and the gene copy numbers to tune circuit symmetry and stability, it does not appear unrealistic to experimentally verify or disprove our findings. Additionally, by letting fluorescent reporters to be under the control of *LacI* and *TetR*, one could explore the bistability states of the [IPTG]-[DOX] plane for different gene and plasmid copy number combinations. If sufficiently automated, this could provide us with diagrams similar to those in Fig. 3.

We further propose that the above (or a similarly designed) bistable switch could be used as a memory component in a genetic measuring device serving for, e.g. monitoring of biochemical environments. If one uses microorganisms for measuring a quantity in a visually accessible environment, it would be possible to employ a microscope to directly observe and measure the expression of a fluorescent reporter. If the measuring location was less accessible, one could imagine extracting the microorganism before performing a measurement. However, there is the possibility that the extraction procedure would perturb the measured values. One solution could be a system that would measure for some time, followed by a period where its detection state is stably maintained while it is extracted and measured. Here is a brief outline of how this could be achieved:

1. A chemical or physical molecular sensor is set to slightly influence the expression of either TetR or LacI.
2. The concentrations of IPTG and DOX are set such that the bistable switch is entered into the fast switching regime, at a location in the phase space where a dilution curve (straight line to the origin) would be completely contained within the bistable region of the [IPTG]-[DOX] plane. Microorganisms containing the above constructs are put into the environment to be measured.
3. As the inducers are diluted, the environment will influence the switch to either be biased toward the TetR- or LacI-dominated states. As the systems enter the disjoint bistable region of the IPTG-DOX plane due to dilution, the population distribution of the switch between the two states will depend on the measured parameters.
4. The microorganisms may now be extracted from the environment without risk of perturbing their states, as in this region of the [IPTG]-[DOX] phase-space the bistable switch is locked into a state by the decreasing inducer concentrations.
5. The states of the switches are measured.

In order to function properly, this approach requires the switch in question to posses the following properties. Firstly, the switch must be bistable in the absence of inhibitors. This is required to preserve the states of the switch throughout the extraction time. Secondly, the bistable region of the switch in inhibitor space must be roughly linear to fully contain a dilution curve. Finally, it is necessary that the bias introduced by the measurement step is small enough not to push the system out of the bistable phase and into monostability when inhibitors are diluted. In the case of the LITR switch, the first requirement is at first glance not met. However, as discussed above, it is possible to tune both the overall and the relative copy numbers of the two repressors to make the switch symmetric and bistable (with no switching) in the absence of inhibitors. The second requirement is related to the overall structure of the two-inhibitor system. In our case, a dilution curve can be plotted within the bistable region and exit into the fast switching region, but this may not necessarily be the case for other switch architectures. Finally, limiting the bias imposed by the molecular sensor on the system performance and properties could be addressed both by modifying the sensor itself, and by increasing the gene copy number in order to widen the bistable region. Overall, the above requirements put strong constraints on the choice of switch components.

## Conclusions

The problem of creating a biomolecular measuring device with memory has previously been addressed by Bhomkar et al., where a recording device was made using a similar switch circuit [19]. However, its principle relied on the inhibition of bacterial division in order to prevent the reporter used for measuring from becoming diluted after the actual measurements were performed. Our method, if practical, does not require such radical precautions. With enough default switch stability, measurements could be recorded after an arbitrary amount of time or an arbitrary number of cell divisions. This stability would be ensured by the fact that each cell stores a binary value, that is relatively easy to preserve. The continuous measured value is then determined from the distribution cells between the two states of the recording device. Using stochastic and deterministic modelling with literature based reaction kinetics we demonstrated that a genetic switch based on the LacI and the TetR transcriptional repressors shows the necessary dynamics to function as such a recording device. We also derived a simplified model for a generalized asymmetric genetic switch, which showed dynamic behaviour similar to the specific case. Overall our modelling provides clues as to how a genetic switch with desirable stability and dynamic properties could be constructed in vivo, whether or not it will be used in a recording device.

## Methods

### Derivation of a reduced set of equations

In the following, we will simplify the equation system in Table 1 and reduce it to two coupled ordinary differential equations in the homodimers for genes 1 and 2: $[\mathrm {P}^{l}_{2}]$. In contrast to previous work [11], we will maintain that the two genes (including their transcription and translation processes) have different properties. This will allow for the study of a more realistic version of this bistable toggle switch, where the two coupled genes have different chemical kinetics.

We begin by making four assumptions: (i) There is only 1 copy of each genetic element, $\sum _{ij}[\mathrm {D}_{ij}^{l}]=1$; (ii) All binding reactions to DNA as well as mRNA elongation are in steady state, $\dot {[\mathrm {D}_{ij}^{l}]} =\dot {[\mathrm {E}^{l}]}=\dot {[\mathrm {M}^{l}]}=0$, where the dot over the variables stands for time derivative; (iii) The concentration of free RNA polymerase is constant, $\dot {[\mathrm {R}]}= 0$; (iv) Dimerization reactions are in steady state, $\dot {[\mathrm {P}^{i}_{2}]} = 0$.

Assumption (i) has the following consequences on the expressions for the repressor molecules, Eqs. (A.1) and (A.2): With only one copy of each gene in each cell, there will be only two binding sites for each repressor. Thus, when the concentration of a repressor molecule ($[\mathrm {P}^{1}_{2}]$ or $[\mathrm {P}^{2}_{2}]$) is low, there is only infrequent binding of the repressor molecules to the operator sequence. When the concentrations of repressor molecules is large, there is likely binding at the operator sequences. However, the 1-2 bound molecules will have a negligible effect on the concentration of the repressors. Consequently, we may discard the effect of repressor-operator binding and rewrite Eqs. (A.1) and (A.2) as

2 $$ \dot{[\mathrm{P}^{i}_{2}]} ~\approx~ k_{i.1}[\mathrm{P}^{i}]^{2} - q_{i.1}[\mathrm{P}^{i}_{2}] - \frac{\gamma_{i.p}}{\sigma_{i}} [\mathrm{P}_{2}^{i}],  $$

with *i*={1,2}.

Regarding the approximation (ii) for the different states of the two genetic elements $\mathrm {D}_{ij}^{k}$, Eqs. (A.5)-(A.16): We first note that, when considering $\mathrm {D}_{ij}^{k}$ the only variables, these twelve equations decouple into six linear equations for each gene (index *k*={1,2}), each set of equations with nullity 1. Solving these two linear equation systems, we express the steady state values $[\mathrm {D}_{ij}^{k}]^{*}$ in terms of $[\mathrm {D}_{00}^{k}]$ as:

3 $$ {}[\mathrm{D}_{10}^{1}]^{*} = \frac{[\mathrm{P}_{2}^{2}]}{K_{1.2}}[\mathrm{D}_{00}^{1}], \quad \, \, \, \, \, \, \, \,[\mathrm{D}_{10}^{2}]^{*} = \frac{[\mathrm{P}_{2}^{1}]}{K_{2.2}}[\mathrm{D}_{00}^{2}],  $$

4 $$ {}[\mathrm{D}_{20}^{1}]^{*} = \frac{[\mathrm{P}_{2}^{2}]^{2} [\mathrm{D}_{00}^{1}]}{K_{1.2} K_{1.4}}, \quad \, \, \, \, \, [\mathrm{D}_{20}^{2}]^{*} = \frac{[\mathrm{P}_{2}^{1}]^{2} [\mathrm{D}_{00}^{2}]}{K_{2.2} K_{2.4}},\\  $$

5 $$ {}[\mathrm{D}_{01}^{1}]^{*} = \frac{[\mathrm{R}]}{K_{1.3}} [\mathrm{D}_{00}^{1}], \qquad \,\,[\mathrm{D}_{01}^{2}]^{*} = \frac{[\mathrm{R}]}{K_{2.3}} [\mathrm{D}_{00}^{2}],  \\  $$

6 $$ {}[\mathrm{D}_{11}^{1}]^{*} = \frac{[\mathrm{R}] [\mathrm{P}_{2}^{2}][\mathrm{D}_{00}^{1}]}{K_{1.2} K_{1.5}}, \,\,\, [\mathrm{D}_{11}^{2}]^{*} = \frac{[\mathrm{R}] [\mathrm{P}_{2}^{1}] [\mathrm{D}_{00}^{2}]}{K_{2.2} K_{2.5}}, \\  $$

7 $$ {}[\mathrm{D}_{21}^{1}]^{*} = \frac{[\mathrm{R}] [\mathrm{P}_{2}^{2}]^{2} [\mathrm{D}_{00}^{1}]}{K_{1.2} K_{1.4} K_{1.7} }, [\mathrm{D}_{21}^{2}]^{*} = \frac{[\mathrm{R}] [\mathrm{P}_{2}^{1}]^{2} [\mathrm{D}_{00}^{2}]}{K_{2.2} K_{2.4} K_{2.7}}   $$

Here, we have introduced the dissociation parameter *K*_*li*_=*q*_*li*_/*k*_*li*_ and made the approximation that (*q*_*li*_+*α*_*lm*_)/*k*_*li*_≈*q*_*li*_/*k*_*li*_=*K*_*li*_ for the dissociation parameters *K*_*l*3_,*K*_*l*5_, and *K*_*l*7_.

For the elongation states E^*l*^, the steady state approximation (ii) together with Eqs. (A.17) and (A.18) gives:

8 $$  \alpha^{\prime}_{lm} [\mathrm{E}^{l}] = \alpha_{lm} ([\mathrm{D}_{01}^{l}]+[\mathrm{D}_{11}^{l}]+\,[\mathrm{D}_{21}^{l}])=f_{l}([\mathrm{P}_{2}^{\varepsilon_{l}}]),  $$

where we have defined *ε*_*l*_=mod(*l*,2)+1. Combining Eq. (8) with Eqs. (3)–(7), we find

9 $$ \begin{aligned} f_{l}([\mathrm{P}_{2}^{\varepsilon_{l}}]) = \alpha_{lm}[\mathrm{D}_{00}^{l}] [\mathrm{R}] \left(\frac{1}{K_{l3}} + \frac{[\mathrm{P}_{2}^{\varepsilon_{l}}]}{K_{l2} K_{l5}} + \frac{[\mathrm{P}_{2}^{\varepsilon_{l}}]^{2}}{K_{l2} K_{l4} K_{l7}}\right) \end{aligned}  $$

We determine the steady-state value of $[\mathrm {D}_{00}^{l}]$ by using assumption (i) and Eqs. (3)–(7), finding

$$\begin{aligned} \frac{1}{[\mathrm{D}_{00}^{l}]} =~ & 1 + \frac{[\mathrm{R}]}{K_{l3}} + \left(1+ \frac{[\mathrm{R}]}{K_{l5}} \right) ~\frac{[\mathrm{P}_{2}^{\varepsilon_{l}}]}{K_{l2}} \\&\quad+ \left(1+ \frac{[\mathrm{R}]}{K_{l7}} \right)~ \frac{K_{l2}}{K_{l4}}~ \left(\frac{[\mathrm{P}_{2}^{\varepsilon_{l}}]}{K_{l2}} \right)^{2} \\ = ~ & (1 + u_{l}^{-1}) \left(1 + \mu_{l} [\mathrm{T}_{\varepsilon_{l}}] ~(1 + r_{l} [\mathrm{T}_{\varepsilon_{l}}]) \right), \end{aligned} $$

where we have introduced the simplifying assumption that *K*_*l*7_=*K*_*l*5_ and the parameters

10 $$ \begin{aligned} s_{l}=\frac{K_{l3}}{K_{l5}}, ~~~~~~ u_{l}=\frac{K_{l3}}{[\mathrm{R}]}, ~~~~~~[\mathrm{T}_{l}]=\frac{[\mathrm{P}_{2}^{l}]}{K_{l2}},\\ r_{l}=\frac{K_{l2}}{K_{l4}}, \mu_{l}=\frac{s_{l} + u_{l}}{1 + u_{l}}. \end{aligned}  $$

Here, the definition of *u*_*l*_ is based on assumption (iii) above, that $\dot {[\mathrm {R}]}= 0$. We will interpret these gene specific parameters as: *s*_*l*_ being a measure of promoter leakage, *u*_*l*_ as the RNAp-promoter dissociation constant scaled by the concentration of free RNAp, [T_*l*_] as the dimensionless concentration of the active repressor molecule (homodimer), and *r*_*l*_ as a measure of cooperativity in the repressor-DNA binding interaction.

Using the requirement of conserved genetic elements (assumption (i)) together with the expressions for $[\mathrm {D}_{ij}^{k*}]$ [Eqs. (3)–(7)], we may rewrite Eq. (9) only as a function of the repressor concentration:

$$\begin{aligned} f_{l}([\mathrm{T}_{l}]) = \frac{1}{(1+u_{l}/s_{l})} \left(1 + \frac{\nu_{l}}{1 + \mu_{l}~ [\mathrm{T}_{\varepsilon_{l}}]~(1 + r_{l}~[\mathrm{T}_{\varepsilon_{l}}])} \right) \end{aligned} $$

where *ν*_*l*_=*u*_*l*_(1−*s*_*l*_)/(*s*_*l*_(1+*u*_*l*_)). Using Eqs. (A.19) and (A.20), the steady state concentration of mRNA [M^*l*^]^∗^ can now be expressed as

11 $$ \begin{aligned} [\mathrm{M}^{l}]^{*}\! =\! \frac{\alpha_{lm}}{\gamma_{lm}(1+u_{l}/s_{l})} \left(1 \,+\, \frac{\nu_{l}}{1 + \mu_{l}~T_{\varepsilon_{l}}~ (1 + r_{l} ~[\mathrm{T}_{\varepsilon_{l}}])} \right). \end{aligned}  $$

By invoking above assumption (iv), that dimerization reactions are fast on the time-scale of translation and transcription and thus can be assumed to be in equilibrium, we may use Eq. (2) to simplify Eqs. (A.3) and (A.4) as:

$$~~\dot{[\mathrm{P}^{l}]} = \alpha_{lp}[\mathrm{M}^{l}] - 2 \frac{\gamma_{lp}}{\sigma_{l}} [\mathrm{P}^{l}_{2}] - \gamma_{lp}[\mathrm{P}^{l}]. $$

Using Eq. (11), we may calculate the change in protein-monomer concentration:

12 $$ \begin{aligned} \dot{p_{l}} &= \lambda_{l} \left(1 + \frac{\nu_{l}}{1 + \mu_{l}~ [\mathrm{T}_{\varepsilon_{l}}]~(1 + r_{l}~[\mathrm{T}_{\varepsilon_{l}}])} \right)\\ &\quad- \left(p_{l} + \frac{2}{\sigma_{l}} [\mathrm{T}_{l}]\right), \end{aligned}  $$

where we have introduced the dimensionless time $\tau =t/\gamma _{lp}^{-1}$, the dimensionless protein-monomer concentration *p*_*l*_= [P_*l*_]/*K*_*l*2_, and the parameters:

$$\beta_{l} =\frac{\alpha_{lp}\alpha_{lm}}{K_{l2} \gamma_{lm} \gamma_{lp}} ~~;~~~~ \lambda_{l}~=~\frac{\beta_{l}}{1+u_{l}/s_{l}}. $$

We interpret *β*_*l*_ as the gene expression efficiency of gene *l* and *λ*_*l*_ as the constitutive synthesis rate of protein *l* during full repression [11]. We may further express Eq. (12) solely in terms of *T*_*l*_ because of assumption (iv): Using the variable change

$$~~~~~p_{l} = \sqrt{[\mathrm{T}_{l}]/\theta_{l}}~~;~~~~~~\dot{p_{l}}=\frac{\dot{[\mathrm{T}_{l}]}}{2\sqrt{\theta_{l}[\mathrm{T}_{l}]}}, $$

where *θ*_*l*_=*K*_*l*2_/*K*_*l*1_, and setting *κ*_*l*_=*σ*_*l*_/*θ*_*l*_=(*σ*_*l*_*K*_*l*1_)/*K*_2_ we find a set of two coupled differential equations shown in Eq. (1).

### Reduced system: derivation of simulation constants

#### Dimerisation of the CI-repressor — *K*_1.1_

In Burz et al. (1994) the dimerization equilibrium constant of the *λ* CI repressor was measured to *K*_eq_=1.8×10^8^ M. Since the *K*_1.1_ parameter is the dissociation constant of this reaction following the relationship,

13 $$ K_{d} = {K_{\text{eq}}}^{-1}  $$

the parameter *K*_1.1_=5.6 nM. Although in the same article they also showed that single-site mutations could cause a change in the dimer dissociation constant up to a value of *K*_1.1_=2.7 M [20]. As this large interval has been shown experimentally it was assumed reasonable to keep using the value used in Ghim and Almaas (2009) [11], where *K*_1.1_=10 nM. The dimerization constant of TetR was assumed to be equal to the one for CI for simplicity, yielding *K*_2.1_=10 nM.

#### Binding of the CI repressor to DNA — *K*_2_

Sauer reported in 1979 that *K*_*d*_=20 nM for CI_2_ dissociation from the O_*R*1_ operator site. While this reference was not available, several others have cited the same value [21, 22]. The value was assumed to be the same for O_*L*1_, the operator site in the P_*L*_-promoter, which gives a *K*_2_=20 nM.

The assumption that *K*_1.2_=*K*_2.2_ is not really valid when comparing CI to the Tet repressor [23]. However, we chose to set them equal to one another for convenience. The parameters were much more carefully chosen for our realistic switch model. This gave *K*_1.2_=*K*_2.2_=20 nM.

#### Co-operative binding to the second operator site — *r*_*l*_

According to Johnson et al. [24] the co-operativity between two CI repressor dimers when binding to O_*R*1_ and O_*R*2_ will decrease the two dissociation constants with a factor of 2 and 12.5, respectively. As the model is made for sequential binding to the two binding sites these two factors are combined to one co-operative factor *r*_2_ = 25. In order to explain the operation of the Tn*10* regulon, regulated by TetR, it is not necessary to include cooperativity to the binding of the repressor to the two repressor sites. However, we assume *r*_2_=*r*_1_ for simplicity, leading to *r*_*l*_=25.

#### Concentration of free RNAp — [R]

The concentration of free RNAp was assumed to be the same as for previous modelling of the *λ*-phage switch [21], [R] =30 nM.

#### Binding of the RNAp to the P_*L*_-promoter — *u*_*l*_

Giladi et al. (1990) reported both the equilibrium constant for RNAp binding to the P_*L*_-promoter and the forward rate constant for the isomerization of closed to open RNAp-promoter complexes. The reported values were 8.94×10^7^ M and 4.38×10^−3^ s^−1^ respectively. These values can be used to estimate that the parameter *K*_1.3_=11.2 nM [25]. The *K*_1.3_-parameter was estimated using the relationship from Eq. (13). As the planned second promoter is P_*Ltet*−*O*_ the value was assumed to be equal for the second promoter as well, which gives *K*_2.3_=11.2 nM. Given our obtained RNAp concentration and Eq. (10), we get *u*_*l*_=3.0.

#### Remaining parameters — *s* and *σ*

The remaining parameters were chosen as in Ghim and Almaas (2009), giving a monomer to dimer lifetime ratio *σ*=10 and the leakage parameter *s*=0.01.

### LITR system: Derivation of rate constants

#### Protein and mRNA decay — *γ*_*p*_,*γ*_*ml*_, and *γ*_*mt*_

The protein degradation in an *E. coli* cell in exponential growth phase has been shown to be dominated by dilution due to cell division [12]. Using a division rate of once every 30 minutes, we obtain *γ*_*p*_=3.9×10^−4^ s^−1^. Using the directly measured lacI mRNA half-life obtained by Bernstein et al. (2002) we determined the *lacI* mRNA degradation constant to be *γ*_*ml*_=3.0×10^−3^ s^−1^. Combining the mRNA half-life of *lacZ* from [26] with the fact that *tetR* mRNA should be degraded three times as fast as that of *lacZ* [27], we obtained *γ*_*mt*_=3.9×10^−3^ s^−1^.

#### Transcription initiation/elongation — *α*_*le*_, and *α*_*lt*_

The transcription initiation rate at both the *lacI* and *tetR* promoters was assumed to be equal to that at the *λP*_*R*_ promoter. We used a value measured at 37 °C [28].

Estimates of the *lacI* transcription elongation rate in *E. coli* range from 28 to 89 bp/s depending on the medium it is grown in [29]. Here we employ a very conservative estimate of 20 bp/s. Since the transcription elongation is carried out by a well-conserved molecular machinery, the same rate can be applied to both the *lacI* and *tetR* genes. Considering the lengths of the mRNA transcripts, we get the respective elongation constants of *α*_*le*_=6.5×10^−2^ s^−1^ and *α*_*te*_=1.1×10^−1^ s^−1^.

#### mRNA translation to protein — *α*_*lt*_, and *α*_*tt*_

The translation rate constant of *lacI* was previously measured to be *α*_*lt*_=1.0×10^−2^ s^−1^ [30]. As no such constant was available for *tetR*, we chose to assume similar translation rate per amino acid. Scaling by the relative length of the *tetR* transcript gave us *α*_*tt*_=1.7×10^−2^ s^−1^.

#### Protein di- and tetramerization — *k*_1_,*q*_1_, and *q*_*l*2_

The association constants of the dimerization and tetramerization reactions were derived from the minimal diffusion limited estimate of the search time of a LacI tetramer for a specific site on the DNA of an *E. coli* cell [16]. Since no data was available for TetR, both TetR and LacI were assumed to dimerize at the same rate *k*_1_=1.5×10^−2^*μ*m^3^. Using the data provided in [31, 32] we derived equilibrium constants for LacI dimerization and tetramerization, from which we obtained the respective dissociation constants (using the value of *k*_1_ above) to be *q*_1_=8.8×10^−5^ s^−1^, and *q*_*l*2_=8.8×10^−8^ s^−1^.

#### Repressor-inducer binding — *k*_*l*5_,*q*_*l*5_,*k*_*t*2_ and *q*_*t*2_

The LacI-IPTG association constant was derived from the rate of dissociation of LacI from DNA after IPTG addition [16], and found to be *k*_*l*5_=1.4×10^−6^*μ*m^3^. The dissociation constant was calculated using *k*_*l*5_ and the respective equilibrium constant [33], giving *q*_*l*5_=8.2×10^−3^ s^−1^. The TetR-doxycycline association and dissociation constants were directly measured by Kedracka et al. (2005). The values were *k*_*t*2_=2.4×10^−4^*μ*m^3^ and *q*_*t*2_=1.9×10^−2^ s^−1^ [34]. The constant *k*_*t*2_ was also used as association constant for the binding of doxycycline to DNA-bound TetR. Similarly *k*_*l*5_ was used for binding of IPTG to DNA-bound LacI (0.5*k*_*l*5_ for reactions with two outcomes). Also *q*_*l*5_ was used as a dissociation constant of IPTG from the DNA-LacI-IPTG complex.

#### DNA-LacI binding — *k*_*l*3_,*q*_*l*3_, and *q*_*l*4_

From Levandoski et al. (1996) we know that the active species of LacI is the tetramer LacI_4_, while the dimer is ineffective at repressing transcription. It is usual that a LacI-controlled promoter has two binding sites. One LacI_4_ is able to bind both sites. Our model goes as follows: If an IPTG molecule is bound to a doubly DNA-bound LacI_4_, then it can displace it from one site but not from the other, but if it binds to a singly DNA-bound LacI_4_, it would either displace it or not, depending on the dimer it binds to. LacI_4_ could exist doubly or singly bound to IPTG or to DNA as well as singly bound to both. Reactions where IPTG displaces LacI_4_ from DNA completely are assumed to be irreversible.

From the single molecule measurements of Elf et al. (2004), we calculated the association constant of LacI_4_ to DNA to be *k*_*l*3_=3.0×10^−3^*μ*m^3^. We assumed that if the tetramer would be bound to one IPTG molecule, it would only have one dimer available for DNA binding, thus reducing this binding rate by a factor of two. We also assumed that if already bound to one binding site on the DNA, it would find the other one much faster, thus here we used 200*k*_*l*3_. Combining *k*_*l*3_ with the equilibrium constant for LacI-DNA binding [32], we found the dissociation rate constant to be *q*_*l*3_=5.4×10^−7^ s^−1^. It is worth mentioning that there exists a variety of different operator sites to which lacI can bind, and some studies suggest over 100 times variability in equilibrium constants between these [18]. We chose the comparatively strong binding site *O*^*sym*^ for our model. It is obvious that the choice of operator would directly affect the simulation results of this specific system. Having a large degree of asymmetry would was in our view better for demonstrational purposes. However, we also tested our circuit with the *O*^1^ operator site [18], yielding very similar results. Finally, we assumed the equilibrium constant to hold for binding to the second site as well, giving *q*_*l*4_=3.0×10^−3^ s^−1^.

#### DNA-TetR binding — *k*_*t*3_ and *q*_*t*3_

The active species of TetR is a dimer TetR_2_. It can bind to a single site on the DNA, however usually TetR-controlled promoters have two TetR binding sites. We assumed that both of the binding sites have the same kinetic parameters, which were fortunately already determined [35]. They are *k*_*t*3_=4.8×10^−3^*μ*m^3^ and *q*_*t*3_=2.1×10^−3^ s^−1^. Inducer doxycycline would irreversibly displace a TetR_2_ from DNA.

#### RNAp binding — *k*_*b*1_,*q*_*b*_,*k*_*lb*2_,*k*_*tb*2_*k*_*lb*3_ and *k*_*tb*3_

The kinetics of *σ*^70^ RNAp binding to *λP*_*R*_ promoter has been determined. We assume that the obtained dissociation rate constant is the same whether repressors are bound to the promoter or not, while the association constant changes depending on the type and number of repressor molecules. The kinetic constants for a free promoter are *k*_*b*1_=4.3×10^−3^*μ*m^3^ and *q*_*b*_=4.5×10^−1^ s^−1^ [28].

We assumed that a singly DNA-bound LacI_4_ (whether bound to IPTG or not) would change the RNAp association rate as much as a bound dimer would. Using expression data for tetramerization mutants [31], we estimated that singly and doubly bound LacI_4_ would yield *k*_*lb*2_=1.2×10^−5^*μ*m^3^ and *k*_*lb*3_=3.6×10^−7^*μ*m^3^ respectively. Using the equilibrium constants for RNAp binding to a TetR-regulated promoter determined by Meier et al. (1988), we found *k*_*tb*2_=5.7×10^−6^*μ*m^3^ and *k*_*tb*3_=8.6×10^−6^*μ*m^3^ [36].

### Stochastic simulation details

The stochastic simulations were performed using the software StochKit2 [37]. This provided us with a fast way of simulating large sets of stochastic equations for extended simulation periods. The stability and switching rate calculations were performed using our own custom software. The criterion for a switch was that the difference between the numbers of the active species had to cross the value of zero. The criterion for bistability was that when initiating the simulation with an excess of either protein, the histograms (of the difference in the number of the active species, e.g. [LacI_4_]−[TetR_2_]) would not intersect. The criterion for monostability was that for both initial conditions >95 *%* of the histogram values would be on the same side. Otherwise the system would be considered jointly bistable (that is, switching is possible within the given simulation time period of 10^7^s). When generating the heat plots in Fig. 3 the simulation time was set to be 10^7^ s, and the number of samples to 10^7^. In Fig. 2 the kinetics was much faster. Therefore the simulation time was set to 10^3^ s while the number of samples was kept the same. In Fig. 4(b-d) each line corresponds to a simulation, with the same time and sampling as in Fig. 3. The Supplementary Materials contains the initial set of equations used to derive the reduced model of the generalized asymmetric switch (Additional file 1).

## Abbreviations

LITR-switch, LacI-TetR genetic switch; IPTG, isopropyl *β*-D-1-thiogalactopyranoside; DOX: doxycycline.

## Additional file

Additional file 1 Equation system describing asymmetric toggle-switch. (PDF 106 kb)
