# Supplementary material for: Hypothetical biomolecular probe based on a genetic switch with tunable symmetry and stability
Source: BMC Syst Biol. 2016 Jun 6;10:39. doi: 10.1186/s12918-016-0279-y (PMC4895904; doi:10.1186/s12918-016-0279-y)
Supplement: Additional file 1 — Equation system describing asymmetric toggle-switch. (PDF 106 kb) [file 12918_2016_279_MOESM1_ESM.pdf]

Equation system describing asymmetric toggle-switch

Using Table 1 and assuming first and second order reaction kinetics, we find the following non-linear coupled ordinary differential equation representation of the circuit in Fig. 1:

$$\dot{[\mathrm{P}_2^1]} = k_{11}[\mathrm{P}^1]^2 - q_{11}[\mathrm{P}_2^1] - \left(\frac{\gamma_{1p}}{\sigma_1} - k_{22}[\mathrm{D}_{00}^2]\right)[\mathrm{P}_2^1] + (q_{22} - k_{24}[\mathrm{P}_2^1])[\mathrm{D}_{10}^2] + q_{24}[\mathrm{D}_{20}^2] \quad (A.1)$$

$$\dot{[\mathrm{P}_2^2]} = k_{21}[\mathrm{P}^2]^2 - q_{21}[\mathrm{P}_2^2] - \left(\frac{\gamma_{2p}}{\sigma_2} - k_{12}[\mathrm{D}_{00}^1]\right)[\mathrm{P}_2^2] + (q_{12} - k_{14}[\mathrm{P}_2^2])[\mathrm{D}_{10}^1] + q_{14}[\mathrm{D}_{20}^1] \quad (A.2)$$

$$\dot{[\mathrm{P}^1]} = \alpha_{1p}[\mathrm{M}^1] + 2q_{11}[\mathrm{P}_2^1] - 2k_{11}[\mathrm{P}^1]^2 - \gamma_{1p}[\mathrm{P}^1] \quad (A.3)$$

$$\dot{[\mathrm{P}^2]} = \alpha_{2p}[\mathrm{M}^2] + 2q_{21}[\mathrm{P}_2^2] - 2k_{21}[\mathrm{P}^2]^2 - \gamma_{2p}[\mathrm{P}^2] \quad (A.4)$$

$$\dot{[\mathrm{D}_{00}^1]} = q_{12}[\mathrm{D}_{10}^1] + (q_{13} + \alpha_{1m})[\mathrm{D}_{01}^1] - (k_{12}[\mathrm{P}_2^2] + k_{13}[\mathrm{R}])[\mathrm{D}_{00}^1] \quad (A.5)$$

$$\dot{[\mathrm{D}_{00}^2]} = q_{22}[\mathrm{D}_{10}^2] + (q_{23} + \alpha_{2m})[\mathrm{D}_{01}^2] - (k_{22}[\mathrm{P}_2^1] + k_{23}[\mathrm{R}])[\mathrm{D}_{00}^2] \quad (A.6)$$

$$\dot{[\mathrm{D}_{10}^1]} = k_{12}[\mathrm{D}_{00}^1][\mathrm{P}_2^2] + q_{14}[\mathrm{D}_{20}^1] + (q_{15} + \alpha_{1m})[\mathrm{D}_{11}^1] - (q_{12} + k_{14}[\mathrm{P}_2^2] + k_{15}[\mathrm{R}])[\mathrm{D}_{10}^1] \quad (A.7)$$

$$\dot{[\mathrm{D}_{10}^2]} = k_{22}[\mathrm{D}_{00}^2][\mathrm{P}_2^1] + q_{24}[\mathrm{D}_{20}^2] + (q_{25} + \alpha_{2m})[\mathrm{D}_{11}^2] - (q_{22} + k_{24}[\mathrm{P}_2^1] + k_{25}[\mathrm{R}])[\mathrm{D}_{10}^2] \quad (A.8)$$

$$\dot{[\mathrm{D}_{20}^1]} = k_{14}[\mathrm{D}_{10}^1][\mathrm{P}_2^2] + (q_{17} + \alpha_{1m})[\mathrm{D}_{21}^1] - (q_{14} + k_{17}[\mathrm{R}])[\mathrm{D}_{20}^1] \quad (A.9)$$

$$\dot{[\mathrm{D}_{20}^2]} = k_{24}[\mathrm{D}_{10}^2][\mathrm{P}_2^1] + (q_{27} + \alpha_{2m})[\mathrm{D}_{21}^2] - (q_{24} + k_{27}[\mathrm{R}])[\mathrm{D}_{20}^2] \quad (A.10)$$

$$\dot{[\mathrm{D}_{01}^1]} = k_{13}[\mathrm{D}_{00}^1][\mathrm{R}] - (q_{13} + \alpha_{1m})[\mathrm{D}_{01}^1] \quad (A.11)$$

$$\dot{[\mathrm{D}_{01}^2]} = k_{23}[\mathrm{D}_{00}^2][\mathrm{R}] - (q_{23} + \alpha_{2m})[\mathrm{D}_{01}^2] \quad (A.12)$$

$$\dot{[\mathrm{D}_{11}^1]} = k_{15}[\mathrm{D}_{10}^1][\mathrm{R}] - (q_{15} + \alpha_{1m})[\mathrm{D}_{11}^1] \quad (A.13)$$

$$\dot{[\mathrm{D}_{11}^2]} = k_{25}[\mathrm{D}_{10}^2][\mathrm{R}] - (q_{25} + \alpha_{2m})[\mathrm{D}_{11}^2] \quad (A.14)$$

$$\dot{[\mathrm{D}_{21}^1]} = k_{17}[\mathrm{D}_{20}^1][\mathrm{R}] - (q_{17} + \alpha_{1m})[\mathrm{D}_{21}^1] \quad (A.15)$$

$$\dot{[\mathrm{D}_{21}^2]} = k_{27}[\mathrm{D}_{20}^2][\mathrm{R}] - (q_{27} + \alpha_{2m})[\mathrm{D}_{21}^2] \quad (A.16)$$

$$\dot{[\mathrm{E}^1]} = \alpha_{1m}([\mathrm{D}_{01}^1] + [\mathrm{D}_{11}^1] + [\mathrm{D}_{21}^1]) - \alpha'_{1m}[\mathrm{E}^1] \quad (A.17)$$

$$\dot{[\mathrm{E}^2]} = \alpha_{2m}([\mathrm{D}_{01}^2] + [\mathrm{D}_{11}^2] + [\mathrm{D}_{21}^2]) - \alpha'_{2m}[\mathrm{E}^2] \quad (A.18)$$

$$\dot{[\mathrm{M}^1]} = \alpha'_{1m}[\mathrm{E}^1] - \gamma_{1m}[\mathrm{M}^1] \quad (A.19)$$

$$\dot{[\mathrm{M}^2]} = \alpha'_{2m}[\mathrm{E}^2] - \gamma_{2m}[\mathrm{M}^2] \quad (A.20)$$

$$\begin{aligned}\dot{[\mathrm{R}]} = {} & q_{13}[\mathrm{D}_{01}^1] + q_{15}[\mathrm{D}_{11}^1] + q_{17}[\mathrm{D}_{21}^1] + q_{23}[\mathrm{D}_{01}^2] + q_{25}[\mathrm{D}_{11}^2] + q_{27}[\mathrm{D}_{21}^2] + \alpha'_{1m}[\mathrm{E}^1] + \alpha'_{2m}[\mathrm{E}^2] \\ & -(k_{13}[\mathrm{D}_{00}^1] + k_{15}[\mathrm{D}_{10}^1] + k_{17}[\mathrm{D}_{20}^1] + k_{23}[\mathrm{D}_{00}^2] + k_{25}[\mathrm{D}_{10}^2] + k_{27}[\mathrm{D}_{20}^2])[\mathrm{R}] \quad (A.21)\end{aligned}$$

Here, square bracket [X] denotes concentration of the chemical species X, and a dot denotes time derivative.
